# Peroral cholangioscopy-guided lithotripsy using a novel thin cholangioscope under balloon enteroscopy for Roux-en-Y anastomosis

**DOI:** 10.1055/a-2299-2477

**Published:** 2024-04-24

**Authors:** Yuki Tanisaka, Masafumi Mizuide, Akashi Fujita, Takahiro Shin, Kei Sugimoto, Ryuhei Jinushi, Shomei Ryozawa

**Affiliations:** 1 183786 Gastroenterology, Saitama Medical University International Medical Center, Hidaka, Japan

Stone extraction using endoscopic retrograde cholangiopancreatography (ERCP) is less invasive than surgical procedures. However, stone extraction in patients with surgically altered anatomy, such as those who have undergone a Roux-en-Y procedure, is challenging. Although balloon enteroscopy is useful for such cases, there is still room for improvement 1 2 . Peroral cholangioscopy (POCS)-guided lithotripsy can aid in the extraction of stones that are difficult to remove 3 4 . However, performing POCS-guided lithotripsy under balloon enteroscopy is difficult because cholangioscopes have an approximate diameter of 10 Fr and cannot pass through the forceps channel of the balloon enteroscope. This report describes a patient with a Roux-en-Y anastomosis who was successfully treated with POCS-guided lithotripsy using a novel thin cholangioscope under balloon enteroscopy.

A 51-year-old woman who had previously undergone a diversion operation and hepaticojejunostomy with Roux-en-Y for congenital biliary dilatation 7 years earlier was referred to our center. Computed tomography revealed large stones in the intrahepatic bile duct ( Fig. 1 ). Consequently, we performed ERCP using a short-type single-balloon enteroscope (SIF-H290; Olympus, Tokyo, Japan) with a working length of 152 cm and a working channel with a diameter of 3.2 mm 1 2 . Additionally, we performed POCS-guided lithotripsy using a thin cholangioscope (eyeMAX; Micro-Tech, Nanjing, China) with a length of 219 cm and diameter of 9 Fr 5 ( Fig. 2 **,** Video 1 ) as complete stone extraction was difficult without POCS. Cholangiography revealed large stones in the intrahepatic bile duct ( Fig. 3 ). Subsequently, POCS was performed using a thin cholangioscope, revealing multiple large stones in the intrahepatic bile duct ( Fig. 4 **a** ). POCS-guided lithotripsy was performed while maintaining a clear field of view ( Fig. 4 **b** , **c** ). Successful stone fragmentation was achieved ( Fig. 4 **d** ), followed by complete stone extraction ( Fig. 5 ).

**Fig. 1:**
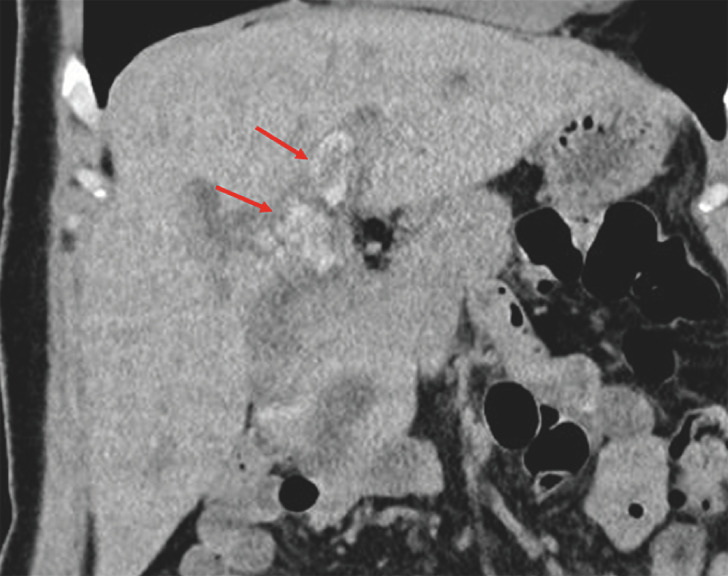
Computed tomography revealed large stones (red arrows) in the intrahepatic bile duct.

**Fig. 2:**
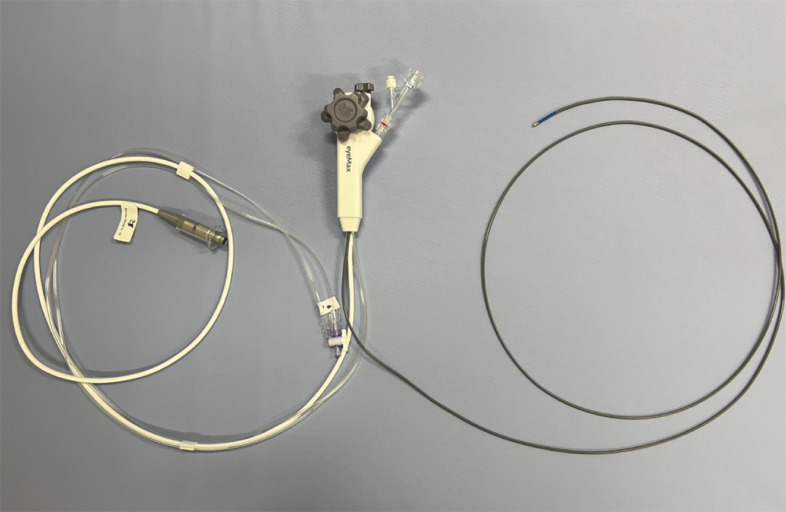
Thin cholangioscope (eyeMAX; Micro-Tech, Nanjing, China) with a length of 219 cm and diameter of 9 Fr.

**Fig. 3:**
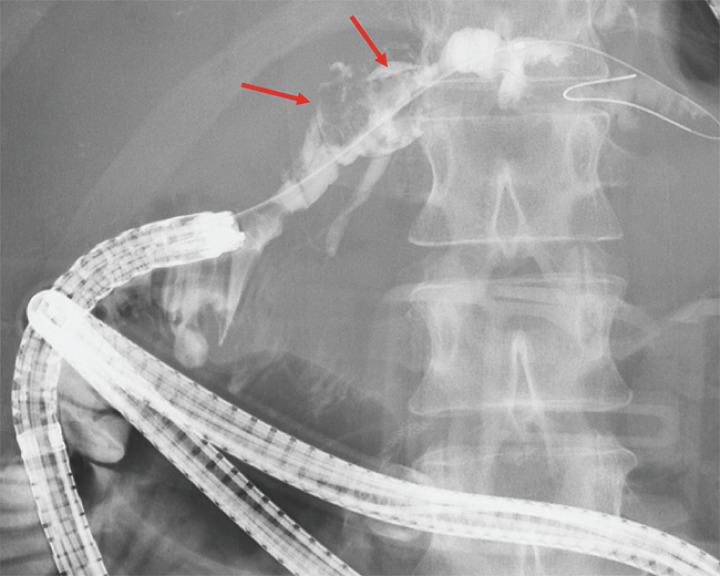
Cholangiography revealed large stones (red arrows) in the intrahepatic bile duct.

**Fig. 4:**
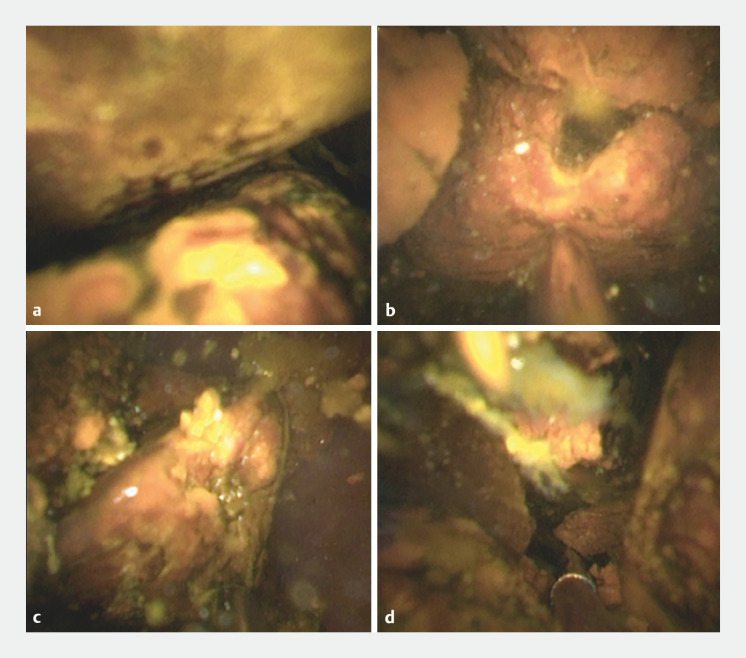
Cholangioscopy findings. **a** Cholangioscopy revealed multiple large stones in the intrahepatic bile duct. **b** , **c** Peroral cholangioscopy-guided lithotripsy is performed while maintaining a clear field of view. **d** Stone fragmentation has been successfully achieved.

**Fig. 5:**
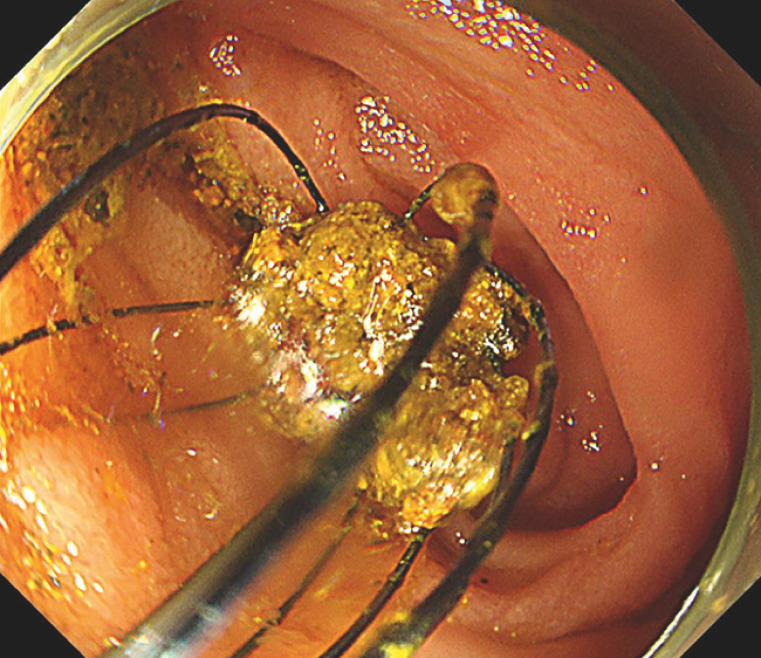
Endoscopy revealed successful stone extraction.

Successful peroral cholangioscopy-guided lithotripsy using a novel thin cholangioscope under balloon enteroscopy in a patient who had previously undergone a Roux-en-Y procedure.Video 1

The thin cholangioscope was effective for POCS-guided lithotripsy even though a balloon enteroscope was used. This novel thin cholangioscope can improve the success rate of stone extraction in patients with a surgically altered anatomy.

Endoscopy_UCTN_Code_TTT_1AR_2AH

## Notes

Endoscopy E-Videos https://eref.thieme.de/e-videos
*E-Videos* is an open access online section of the journal *Endoscopy* , reporting on interesting cases and new techniques in gastroenterological endoscopy. All papers include a high-quality video and are published with a Creative Commons CC-BY license. Endoscopy E-Videos qualify for HINARI discounts and waivers and eligibility is automatically checked during the submission process. We grant 100% waivers to articles whose corresponding authors are based in Group A countries and 50% waivers to those who are based in Group B countries as classified by Research4Life (see: https://www.research4life.org/access/eligibility/ ). This section has its own submission website at https://mc.manuscriptcentral.com/e-videos .
